# Supplementary material for: Real-time classification of longitudinal conveyor belt cracks with deep-learning approach
Source: PLoS One. 2023 Jul 20;18(7):e0284788. doi: 10.1371/journal.pone.0284788 (PMC10358885; doi:10.1371/journal.pone.0284788)
Supplement: Attachment — Submitted filename: Dwivediet al_BelconAI_Rebuttal Letter_12112022.docx [file pone.0284788.s001.docx]

**Uttam Kumar Dwivedi,**

[uttamdwivedi04@gmail.com](mailto:uttamdwivedi04@gmail.com)

University of Tokyo, Tokyo Japan

December 11^th^, 2022

Dear Brij Bhooshan Gupta,

*Academic Editor*,

PLOS ONE.

Warm regards. I sincerely thank you for informing the referees’ reports on our initial article titled “*Realtime detection and categorization of longitudinal cracks in conveyor belt using deep-learning approach*” (**PONE-D-22-23772).** I thank you and both the referees for their valuable time and constructive remarks. Following their comments to the letters, we have significantly modified the article presenting a completely new method of conveyor belt damage identification. Please find below the answers to all the questions raised by the referees on a point-by-point format.

**Reviewer: 1**

***Comment:* I found the paper a little difficult to read because of the inconsistent use of good language and the ambiguous way the topic was presented. Particularly in this work, the presentation quality needs to be improved. By fully rewriting the content, an accomplished English-language author may significantly improve this document. I have the following ideas to improve this essay:**

**Reply:** Thank you very much for your constructive criticism and suggestions. We have modified our manuscript in accordance with your suggestions.

**-Describe your contribution better.**

Reply: The corrections have been incorporated and highlighted in the revised manuscript.

**-The literature review is sufficient, but the authors should organise them more effectively.**

Reply: Following your suggestion, we have added a new literature review section in the article to make the manuscript more coherent.

**-Review the following articles to strengthen the paper's technical foundation:**

**Handling Data Scarcity Through Data Augmentation in Training of Deep Neural Networks for 3D Data Processing, Improved Semantic Representation Learning by Multiple Clustering for Image-Based 3D Model Retrieval, Optimization of the Wake-Up Scheduling Using a Hybrid of Memetic and Tabu Search Algorithms for 3D-Wireless Sensor Networks, Unobtrusive academic emotion recognition based on facial expression using rgb-d camera using adaptive-network-based fuzzy inference system (ANFIS),Accelerating 3D medical volume segmentation using GPUs**

Reply: We have carefully reviewed the mentioned papers and cited them in our revised manuscript.

**– Carefully correct all errors in this paper.**

Reply: We have carefully proofread our manuscript for submission.

**-Improve the paper's connections and overall flow.**

Reply: We have revised our manuscript to present a clear outlook and subject flow.

**Reviewer: 2**

***Comment:* The Author has made an effort to proposed Realtime detection and categorization of longitudinal cracks in conveyor belt using deep-learning approach.**

**Title is needed to relook and make it more appropriate in the view of contribution. Further, in the abstract, author relook its flow and highlight the key contributions.**

**• Paper should be strictly according to the journal template.**

Reply: We have revised our manuscript as per the journal template.

**• Improve the figure quality.**

Reply: We have improved figure quality.

**• Add some comparison results and discussion.**

Reply: We have added comparison results and discussions.

**• Give a comparison from previous work.**

Reply: We have added and highlighted in our main text.

**• Check for grammar and spellings.**

Reply: We have carefully proofread for possible grammar and spelling errors.

**• Literature review section need to be added.**

Reply: We have added and highlighted Literature review section.

**• Result and discussion part is required to be improved.**

Reply: We have improved results and discussion sections to give better prospects to the reader.

**• State the novelty of your work.**

Reply: We have highlighted the novelty in the revised text.

**• The overall manuscript should be checked for typos, syntax, and grammar to improve the quality of content flow and presentation**

Reply: The typos, syntax and grammar have been improved.

**• More Number of References Should be added in your article**

Reply: We have cited more references.

I once again sincerely thank you and both the referees for their time, effort and constructive suggestions. Addressing their concerns, we have thoroughly revised the manuscript and we hope it meets the high standard of *PLOS One journal*. Thank you very much.

Sincerely yours,

Uttam Kumar Dwivedi
