# Supplementary material for: Real-time classification of longitudinal conveyor belt cracks with deep-learning approach
Source: PLoS One. 2023 Jul 20;18(7):e0284788. doi: 10.1371/journal.pone.0284788 (PMC10358885; doi:10.1371/journal.pone.0284788)
Supplement: Attachment — Submitted filename: Dwivediet al_BelconAI_Rebuttal Letter_03222023.docx [file pone.0284788.s002.docx]

**Uttam Kumar Dwivedi,**

[uttamdwivedi04@gmail.com](mailto:uttamdwivedi04@gmail.com)

University of Tokyo, Tokyo Japan

March 22^nd^, 2023

Dear Brij Bhooshan Gupta,

*Academic Editor*,

PLOS ONE.

Warm regards. I sincerely thank you for informing the referees’ reports on our initial article titled “*Real-time classification of longitudinal conveyor belt cracks with deep-learning approach*” (**PONE-D-22-23772).** I thank you and both the referees for their valuable time and constructive remarks. Following their comments to the letters, we have significantly modified the article presenting a completely new method of conveyor belt damage identification. Please find below the answers to all the questions raised by the referees on a point-by-point format.

**Reviewer: 1**

***Comment:* I found the topic of your research to be interesting and relevant. However, I have identified some issues that require your attention. Firstly, I recommend that you expand your literature review to include more recent and relevant sources. i suggest a few like: An edge-AI based forecasting approach for improving smart microgrid efficiency, A multimodal, multimedia point-of-care deep learning framework for COVID-19 diagnosis, Service orchestration of optimizing continuous features in industrial surveillance using big data based fog-enabled internet of things, A novel approach for phishing URLs detection using lexical based machine learning in a real-time environment**

**Also, ensure that your introduction clearly and effectively contextualizes your research question. Furthermore, I noticed some inconsistencies in the data presented, which need to be addressed. Please review your results and ensure that they are presented clearly.**

**Finally, I have observed some minor issues with grammar and syntax that need to be addressed:**

**Reply:** Thank you very much for your constructive criticism and suggestions. We understand your concerns regarding the expansion of literature review with the mentioned articles. However the mentioned articles don’t fit in our current discussion and are out of context to our study of damage detection in conveyor belts or in construction sites.

Throughout our introduction we focused on addressing the research question: “How can edge AI-based deep learning framework be used to detect and track damages on long conveyor belts in real-time without halting the mucking process and ensure safety and productivity at mountain tunnel construction sites?” We have clearly mentioned this in our revised manuscript and highlighted.

Also, we have followed and addressed your earlier comments, which mentioned the literature review being sufficient and in need of better organizations. So at this stage we request you to kindly consider the manuscript literature section as it is. This is in-sync with the Reviewer 2.

We have carefully analysed our method statement and results. We are quite confident on our presentation. However, if you would kindly be more precise on which part of data presentation contains inconsistencies, we are ready to address and answer.

We have carefully proofread the manuscript and checked it through the professional software as well for spellings and grammar. We are happy for further proofread formalities if mentioned specifically.

**Reviewer: 2**

***Comment:* The Author has incorporated all the suggestions given in first review with respect to the made an effort to proposed Realtime detection and categorization of longitudinal cracks in conveyor belt using deep-learning approach**

**Reply:** We are delighted to receive positive response. We are grateful for all your efforts.

I once again sincerely thank you and both the referees for their time, effort and constructive suggestions. Addressing their concerns, we have thoroughly revised the manuscript and we hope it meets the high standard of *PLOS One journal*. Thank you very much.

Sincerely yours,

Uttam Kumar Dwivedi
